# Oropharyngeal squamous cell carcinoma: radiomic machine-learning classifiers from multiparametric MR images for determination of HPV infection status

**DOI:** 10.1038/s41598-020-74479-x

**Published:** 2020-10-16

**Authors:** Chong Hyun Suh, Kyung Hwa Lee, Young Jun Choi, Sae Rom Chung, Jung Hwan Baek, Jeong Hyun Lee, Jihye Yun, Sungwon Ham, Namkug Kim

**Affiliations:** 1 Department of Radiology and Research Institute of Radiology, University of Ulsan College of Medicine, Asan Medical Center, 86 Asanbyeongwon-Gil, Songpa-Gu, Seoul, 05505 Republic of Korea; 2 Department of Medicine, University of Ulsan College of Medicine, Asan Medical Center, Seoul, Republic of Korea; 3 Department of Biomedical Engineering, Asan Medical Institute of Convergence Science and Technology, University of Ulsan College of Medicine, Asan Medical Center, Seoul, Republic of Korea; 4 Department of Convergence Medicine, University of Ulsan College of Medicine, Asan Medical Center, 86 Asanbyeongwon-Gil, Songpa-Gu, Seoul, 05505 Republic of Korea

**Keywords:** Biotechnology, Cancer, Computational biology and bioinformatics

## Abstract

We investigated the ability of machine-learning classifiers on radiomics from pre-treatment multiparametric magnetic resonance imaging (MRI) to accurately predict human papillomavirus (HPV) status in patients with oropharyngeal squamous cell carcinoma (OPSCC). This retrospective study collected data of 60 patients (48 HPV-positive and 12 HPV-negative) with newly diagnosed histopathologically proved OPSCC, who underwent head and neck MRIs consisting of axial T1WI, T2WI, CE-T1WI, and apparent diffusion coefficient (ADC) maps from diffusion-weighted imaging (DWI). The median age was 59 years (the range being 35 to 85 years), and 83.3% of patients were male. The imaging data were randomised into a training set (32 HPV-positive and 8 HPV-negative OPSCC) and a test set (16 HPV-positive and 4 HPV-negative OPSCC) in each fold. 1618 quantitative features were extracted from manually delineated regions-of-interest of primary tumour and one definite lymph node in each sequence. After feature selection by using the least absolute shrinkage and selection operator (LASSO), three different machine-learning classifiers (logistic regression, random forest, and XG boost) were trained and compared in the setting of various combinations between four sequences. The highest diagnostic accuracies were achieved when using all sequences, and the difference was significant only when the combination did not include the ADC map. Using all sequences, logistic regression and the random forest classifier yielded higher accuracy compared with the that of the XG boost classifier, with mean area under curve (AUC) values of 0.77, 0.76, and 0.71, respectively. The machine-learning classifier of non-invasive and quantitative radiomics signature could guide the classification of the HPV status.

## Introduction

Human papillomavirus (HPV) status is a dependable and independent prognostic factor in patients with oropharyngeal squamous cell carcinoma (OPSCC). Patients with HPV-positive OPSCC have better survival rates than patients with HPV-negative OPSCC^1^. Because of differences in the oncogenesis, epidemiology, and prognosis; the eighth edition of the American Joint Committee on Cancer (AJCC) tumour-node-metastasis staging system classifies OPSCC into HPV-positive and HPV-negative tumours^2^. Therefore, the preoperative differentiation between HPV-positive and HPV-negative OPSCC is critical for patient management as well as prognosis^3^.

The distinct oncogenesis of HPV-positive OPSCC results in characteristic histopathology^4,5^, perfusion, and diffusion parameters, which are related to the angiogenesis and cellularity of the tumour. Several studies have reported diagnosis of the HPV status in patients with OPSCC using preoperative computed tomography (CT) or magnetic resonance (MR) imaging^6–8^. HPV-positive OPSCC tends to exhibit cystic cervical lymph node metastasis^6–8^ and primary tumours with well-defined borders and an exophytic appearance^7^. Recent studies reported that diffusion-weighted imaging (DWI) may help predict HPV status in patients with OPSCC, as HPV-positive OPSCC reveals a low mean apparent diffusion coefficient (ADC) compared with HPV-negative OPSCC^9–11^. Furthermore, a histogram analysis based on dynamic contrast-enhanced MR image showed significantly higher K_ep_ kurtosis values and lower V_e_ min values in patients with p16-positive OPSCC^12^. Recently, several published studies had addressed the prediction of HPV status employing a CT-based radiomics approach; however, their diagnostic performance was moderate (area under the curve; AUC, 0.75–0.80)^13–15^. To date, no studies reported on the application of radiomic machine-learning classifiers on multiparametric MR images to predict HPV status in patients with OPSCC. Therefore, we hypothesise that pre-treatment multiparametric MR image combined with DWI could predict HPV status accurately employing radiomic machine-learning classifiers in patients with OPSCC.

## Results

### Study population and imaging dataset

Of the 70 consecutive patients with OPSCC, 10 were excluded owing to unknown HPV status (n = 4), post-treatment MR images (n = 4), and loss of MR image data (n = 2). Finally, 60 consecutive patients with OPSCC were enrolled in this study (Table 1). Forty-eight patients (80%) were HPV-positive, and 12 patients (20%) had HPV-negative OPSCCs. The median age was 59 years (range: 35 to 85 years), and 83.3% of the patients were male. The imaging data were randomised into a training set (40 MR images containing 32 HPV-positive and 8 HPV-negative OPSCC) and a test set (20 MR images containing 16 HPV-positive and 4 HPV-negative OPSCC) in each fold.

**Table 1:** Baseline characteristics of the included patients.

Sequence	HPV+ oropharyngeal cancer (n = 48)	HPV− oropharyngeal cancer (n = 12)
Age (mean ± SD)	60.6 ± 8.6	59.4 ± 15.7
Male:female	39:9	11:1
**Subsite of origin, no (%)**		
Tonsil	34 (71%)	6 (50%)
Base of tongue	8 (17%)	3 (25%)
Posterior pharyngeal wall	2 (4%)	3 (25%)
Soft palate	1 (2%)	0 (0%)
No evidence of primary tumor	3 (6%)	0 (0%)
**T stage**^**a**^**, no (%)**		
0	3 (6%)	0 (0%)
1	7 (15%)	0 (0%)
2	20 (42%)	4 (33%)
3	3 (6%)	3 (25%)
4	15 (31%)	5^b^ (42%)
**N stage**^**a**^**, no (%)**		
0	7 (15%)	2 (17%)
1	31 (65%)	0 (0%)
2	10 (21%)	9^c^ (75%)
3	0 (0%)	1^d^ (8%)
**M stage**^**a**^**, no (%)**		
0	47 (98%)	11 (92%)
1	1 (2%)	1 (8%)

*SD* standard deviation.

^a^TNM staging was based on AJCC 8th edition.

^b^Five patients were T4a.

^c^Five patents were N2b and four patients were N2c.

^d^One patient was N3b.

### Selected features

**Figure 1:**
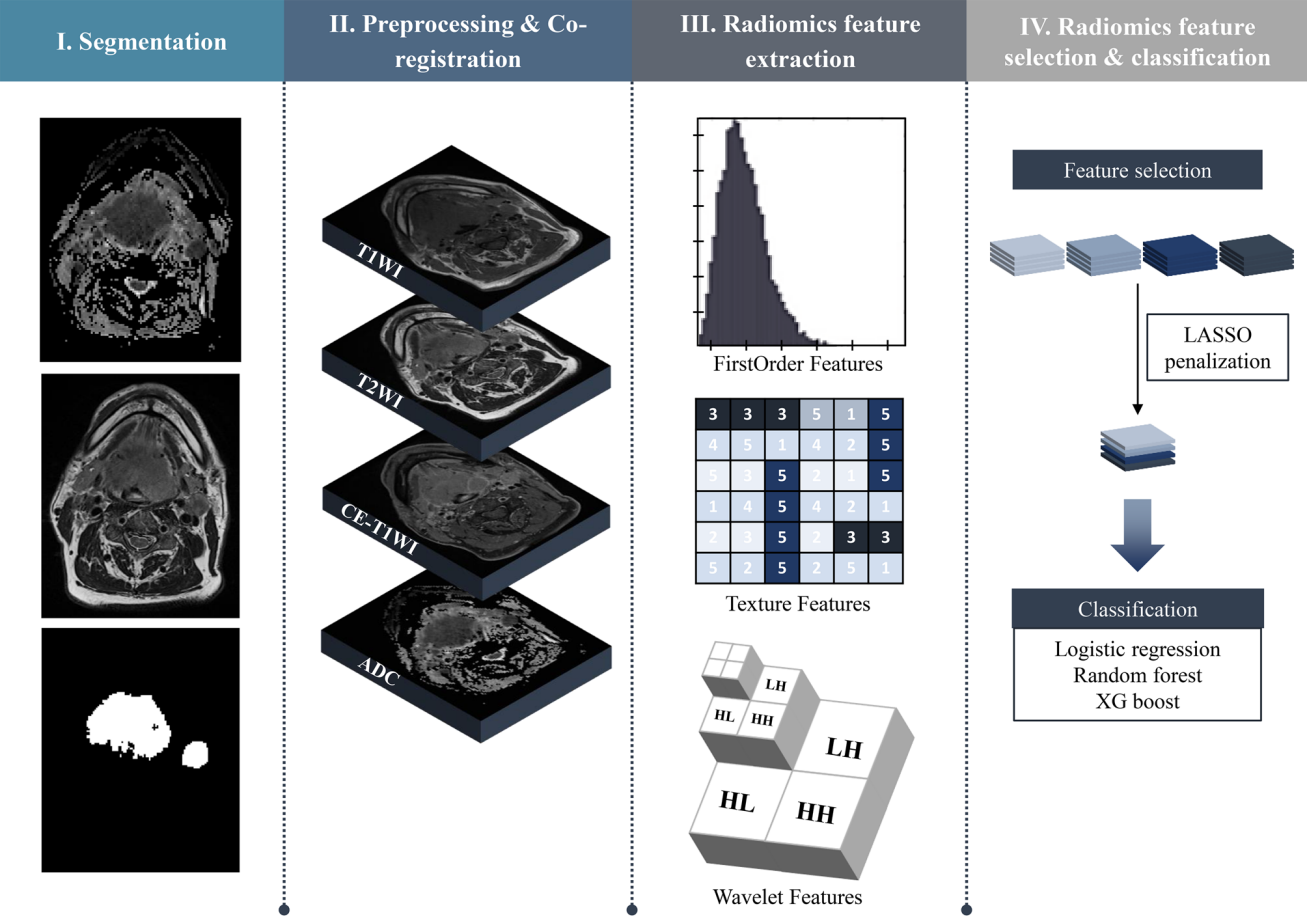
Flowchart of the radiomic machine-learning classifier.

**Figure 2:**
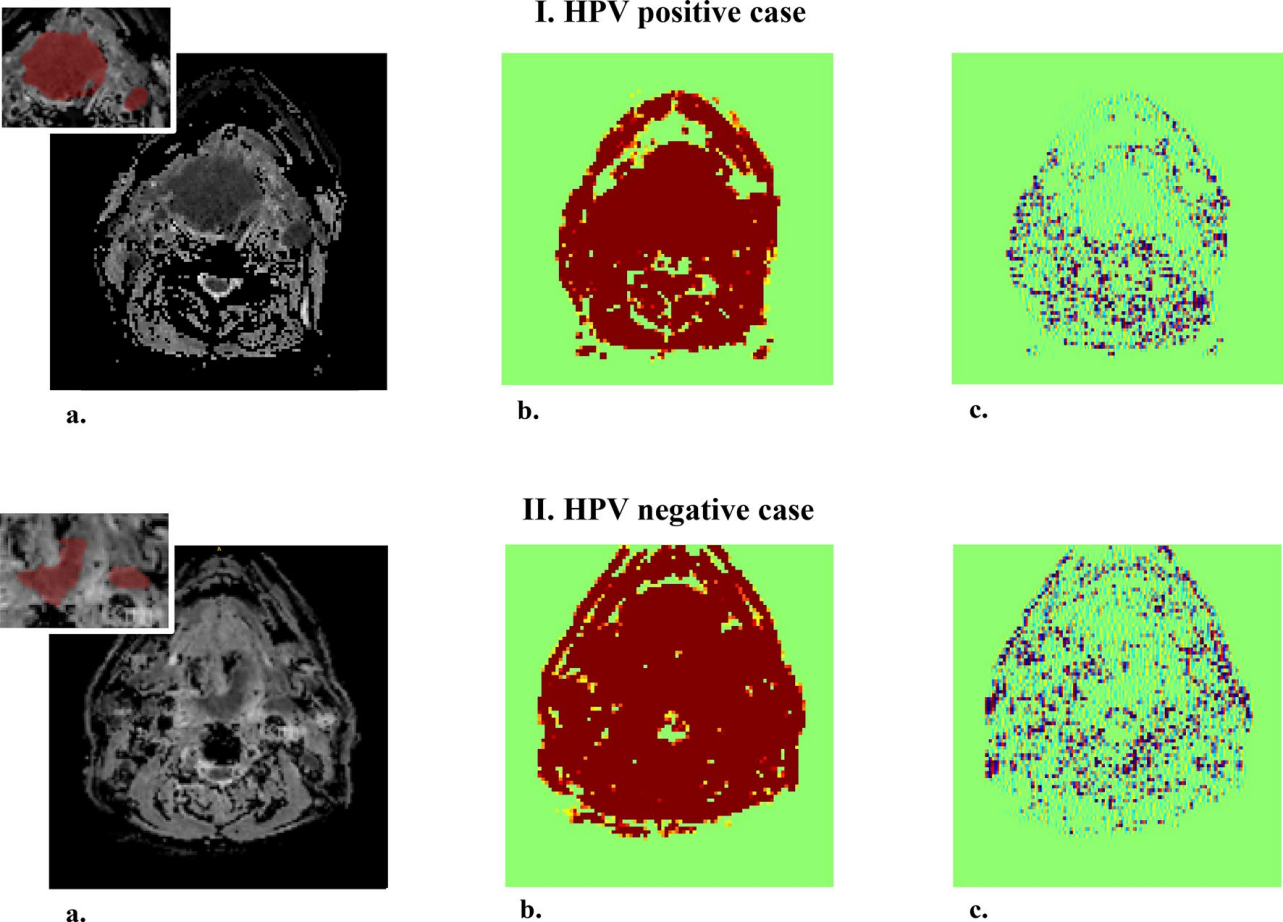
Example of the original apparent diffusion coefficient (ADC) map and its 3D wavelet-transformed image for each human papillomavirus (HPV)-positive and HPV-negative case. (**a**) Original ADC map. (**b**) 3D wavelet-transformed image of ‘LLL’. (**c**) 3D wavelet-transformed image of ‘HLH’.

The study design is shown in Fig. 1. Linear regression with the least absolute shrinkage and selection operator (LASSO) penalty was performed in each cross-validation fold. The average number of selected features with the best classification performance was 221, using four MR sequences, namely, the axial T1-weighted imaging (T1WI), fat-suppressed T2-weighted imaging (T2WI), axial fat-suppressed contrast-enhanced T1-weighted imaging (CE-T1WI), and ADC maps from DWI. Table 2 shows the seven top-performing features, which were sorted based on the frequency of selection in the 60 experiments multiplied by the sum of the LASSO coefficients (weights) in each validation. Six out of the seven features extracted from ADC maps and one feature extracted from the T1WI sequence were selected. Four of these features were wavelet-transformed features. Supplementary Figure 1 illustrates the different ranges of the seven features for HPV-positive and HPV-negative cases in the whole dataset. Six out of seven features exhibited statistically significant differences between the two groups. Figure 2 shows an example of the original ADC map and its wavelet-transformed images of ‘LLL’ and ‘HLH’, where the features with the highest values of the sum of LASSO coefficients are found. The list of the top five selected features from each sequence and their various combinations are described in Supplementary Table 1. In the additional experiment comparing features extracted from primary tumour (T) and nodal (N) volumes delineated on ADC maps, four out of the five top-performing features from T volumes exhibited significant differences between the HPV-positive and HPV-negative group, whereas the features extracted from N volumes did not exhibit significant differences (Supplementary Figure 2).

**Table 2:** Top 7 features from four MR sequences.

Sequence	Wavelets	Class	Variables	Frequency	Sum_Coef*	Freq^a^ Sum_Coef
ADC	LLL	GLCM_dist_2	Entropy_std	58/60 (0.96)	2.547	2.462
T1	Original	GLCM_dist_1	Autocorrelation_std	52/60 (0.86)	1.494	1.295
ADC	HLH	GLCM_dist_2	Correlation_std	45/60 (0.75)	1.368	1.026
ADC	LLH	GLCM_dist_1	Homogeneity1_std	47/60 (0.78)	1.230	0.964
ADC	Original	GLCM_dist_3	Entropy_std	44/60 (0.73)	0.887	0.651
ADC	HHH	GLCM_dist_3	Correlation	40/60 (0.66)	0.950	0.633
ADC	Original	GLCM_dist_1	Difference variance	55/60 (0.91)	0.654	0.599

*ADC*  apparent diffusion coefficient, *T1WI*  T1-weighted imaging, *GLCM*  gray-level co-occurrence matrix.

^a^Sum of LASSO coefficients (= weights).

### Comparing accuracies between sequences

The overall accuracy was increased by adding another MR sequence regardless of the types of classifiers. Table 3 lists the mean AUCs with standard deviations of each sequence and their combinations obtained by three different classifiers. The highest accuracy was achieved using four MR sequences. Upon comparison of each combination and all sequences as a reference for each classifier, the inclusion of all sequences yielded a significantly superior performance to that obtained using three sequences or less, exclusively when the combination did not include the ADC map. There were no significant differences between using three sequences or less while including the ADC map and using all sequences with a random forest and XG boost classifier.

**Table 3:** Classification accuracies between various combinations of sequences.

Sequence	No. of selected features	AUC					
		Logistic regression	*P* value	Random forest	*P* value	XG boost	*P* value
ADC	166	0.72 ± 0.11	.016	0.76 ± 0.11	.456	0.69 ± 0.11	.240
T1WI	160	0.42 ± 0.15	< .001	0.45 ± 0.13	< .001	0.43 ± 0.17	< .001
T2WI	156	0.47 ± 0.13	< .001	0.52 ± 0.13	< .001	0.50 ± 0.12	< .001
CE-T1WI	165	0.55 ± 0.12	< .001	0.54 ± 0.13	< .001	0.59 ± 0.15	< .001
ADC + T1WI	190	0.69 ± 0.12	< .001	0.74 ± 0.11	.165	0.71 ± 0.11	.393
ADC + T2WI	196	0.72 ± 0.11	.020	0.73 ± 0.11	.141	0.69 ± 0.11	.113
ADC + CE-T1WI	193	0.76 ± 0.11	.357	0.76 ± 0.12	.495	0.71 ± 0.14	.481
T1WI + T2WI	185	0.48 ± 0.15	< .001	0.46 ± 0.13	< .001	0.44 ± 0.16	< .001
T1WI + CE-T1WI	200	0.56 ± 0.13	< .001	0.56 ± 0.14	< .001	0.51 ± 0.14	< .001
T2WI + CE-T1WI	191	0.52 ± 0.13	< .001	0.54 ± 0.14	< .001	0.51 ± 0.14	< .001
ADC + T1WI + T2WI	210	0.69 ± 0.14	.003	0.73 ± 0.11	.167	0.69 ± 0.12	.229
ADC + T1WI + CE-T1WI	211	0.76 ± 0.11	.316	0.74 ± 0.11	.186	0.71 ± 0.12	.482
ADC + T2WI + CE-T1WI	212	0.75 ± 0.11	.173	0.74 ± 0.11	.181	0.70 ± 0.12	.373
T1WI + T2WI + CE-T1WI	213	0.53 ± 0.15	< .001	0.54 ± 0.15	< .001	0.50 ± 0.14	< .001
All	221	0.77 ± 0.12	*Ref*	0.76 ± 0.12	*Ref*	0.71 ± 0.12	*Ref*

Average results ± standard deviations are reported.

*AUC* area under the curve, *ADC* apparent diffusion coefficient, *T1WI* T1-weighted imaging, *T2WI* fat-suppressed T2-weighted imaging, *CE-T1WI* fat-suppressed contrast-enhanced T1-weighted imaging.

### Comparing accuracies between machine-learning classifiers

**Figure 3:**
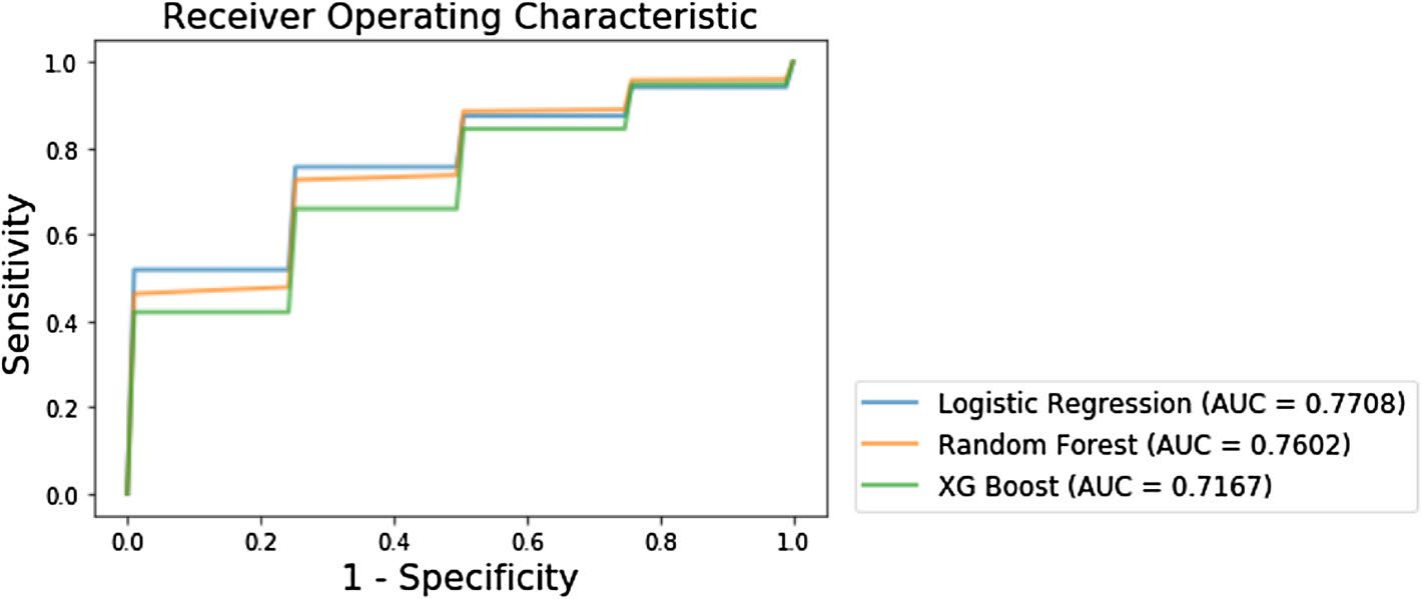
Results of the receiver operating characteristic curve analysis of three classifiers.

The mean AUCs of logistic regression, random forest, and XG boost classifier were 0.77 ± 0.12 (95% confidence interval [CI] 0.50 to 0.96), 0.76 ± 0.12 (95% CI 0.47 to 0.97), and 0.71 ± 0.12 (95% CI 0.50 to 0.93), respectively, when using selected features from all sequences (Fig. 3). The logistic regression classifier yielded the highest value of the mean AUC, which was not significantly superior to that exhibited by the random forest classifier (P value = 0.338), while demonstrating performance superior to that of the XG boost classifier (P value = 0.009). The average sensitivity and specificity were 0.71 (95% CI 0.31 to 0.97) and 0.72 (95% CI 0.50 to 1.00) in the logistic regression classifier, 0.70 (95% CI 0.33 to 0.93) and 0.72 (95% CI 0.50 to 1.00) in the random forest classifier, and 0.62 (95% CI 0.21 to 0.90) and 0.65 (95% CI 0.25 to 1.00) in the XG boost classifier, respectively, as shown in Table 4.

**Table 4:** Results of the ROC curve analysis of 3 models.

Classifiers	AUC	Sensitivity	Specificity
Logistic regression	0.77 (0.50, 0.96)	0.71 (0.31, 0.97)	0.72 (0.50, 1.00)
Random forest	0.76 (0.47, 0.97)	0.70 (0.33, 0.93)	0.72 (0.50, 1.00)
XG boost	0.71 (0.50, 0.93)	0.62 (0.21, 0.90)	0.65 (0.25, 0.10)

Unless otherwise specified, data are averages, with 95% confidence interval in parentheses.

*ROC* receiver operator characteristic, *AUC* area under the curve, *CI* confidence interval.

## Discussion

In the present study, we extracted quantitative image features from multiparametric MR sequences in OPSCC patients and developed machine-learning classifiers following a feature reduction to identify the HPV infection status. Our results show that the logistic regression classifier (0.77 ± 0.12) and the random forest classifier (0.76 ± 0.12) demonstrate higher values of the mean AUC compared with those exhibited by the XG boost classifiers (0.71 ± 0.12). The average sensitivity and specificity in the logistic regression classifier were 0.71 and 0.72, respectively. This radiomic signature of HPV status can be used to develop non-invasive tools for discriminating OPSCC patients.

Increasing evidence suggests that radiomics, a method that non-invasively extracts quantitative information from medical images, can be used to characterize intra-tumoral heterogeneity^16–19^. Previous exploratory studies indicate a correlation between the HPV infection status and CT-based radiomic signature in head and neck squamous cell carcinoma (HNSCC)^13–15,20^. These studies reported AUC values that ranged from 0.70 to 0.86. Although most radiomics studies for classifying the HPV status are based on CT, Ravanelli et al. investigated the correlation between MR imaging texture features and HPV status in OPSCC^9^. The authors developed a simple predictive model based on mean ADC values and smoking status that yielded an AUC of 0.944. In the present study, we developed a tool for classifying the HPV status using radiomic features from multiparametric MR images and machine-learning classifiers with an AUC of 0.77.

Recent studies have addressed whether the ADC-histogram analysis can be used to identify different histopathological features in HNSCC^9,21,22^. According to de Perrot et al., diffusion phenotypes based on the histogram analysis of ADC values reflect distinct degrees of tumour heterogeneity in HPV-positive and HPV-negative HNSCCs^21^. It has been shown that the mean and median ADCs are significantly lower, whereas excess kurtosis and skewness are significantly higher in HPV-positive tumours than in HPV-negative tumours. In their study, HPV-positive tumours exhibit leptokurtic right-skewed histograms, which correspond to homogeneous tumours with densely packed cells, a scant stromal component, and scattered comedonecrosis. Meanwhile, HPV-negative tumours exhibit symmetric normally distributed ADC histograms, which correspond to heterogeneous tumours with variable cellularity, a high stromal component, keratin pearls, and necrosis. Meyer et al. investigated the correlation of ADC values with prognostically relevant histopathologic parameters, including the expression of Hif1-alpha, VEGF, EGFR, p53, p16, and Her 2^22^. They found that ADC histogram reflects different histopathological features in HNSCC, and associations between ADC histogram parameters and histopathology depend on the p16 status. In this study, features extracted from ADC maps were attributed the highest weight after LASSO regression, and they were mostly included in the top-performing features.

Recent studies found that the radiomics signature from multiparametric MR images achieved higher prognostic accuracies compared with a single MR sequence^23–26^. In the present study, using four MR sequences yielded the highest classification accuracy. However, the difference between using four sequences and three or less sequences was significant only in cases not including ADC maps. The selected features after LASSO regression from four MR sequences included features from all MR sequences, whereas features from the ADC map comprised a large percent of top-performing features. Considering a small sample size and imbalance of HPV status in this study, further studies might be needed to confirm whether combining multiple MR sequences enables the detection of more detailed differences between HPV-positive and HPV-negative tumours.

Machine-learning models have rapidly improved in the past few years. Radiomics is an emerging field for machine-learning that allows the conversion of radiologic images into mineable high-dimensional data^24,27–30^. Only few studies investigated the effect of different feature selections and machine-learning classification methods on radiomic features^27,30^. In these studies, the random forest classifier had the highest prognostic performance for diagnosing cancers from benign tumours. Further, Parmar et al. observed that a generalised linear model exhibits a high prognostic performance in HNSCC and non-small-cell lung cancer types, whereas it shows low stability for HNSCC^27^. The present study compared three machine-learning classifiers including the logistic regression, random forest, and XG boost model. The logistic regression classifier and random forest classifier demonstrated performance superior to that of the XG boost classifier. The most plausible reason is that the final selected features are highly discriminative in their classification of HPV status, which proves to be most suitable for the logistic regression classifier. However, considering that logistic regression models generally perform better for smaller data sets, compared with tree induction models, and are prone to overfitting^31,32^, further validation with large samples might be needed.

Our study has several limitations. First, it is a retrospective study performed on a relatively small sample with a highly imbalanced dataset for machine-learning (n = 60). Repeated cross-validation and feature selection using the LASSO regression were applied to mitigate the risk of overfitting in this situation. Second, it remains to be validated whether our radiomics signature can be applied to different MR systems, imaging protocols, and software platforms. Therefore, multi-centre studies with large samples and a prospective study design are required to evaluate the true predictive value of the radiomics signature. Third, the regions-of-interest (ROIs) in the tumours were manually delineated based on ADC maps, which tend to be affected by movement artefacts such as breathing and swallowing, along with frequent susceptibility artefacts from the air-tissue interface. Furthermore, the stability analysis, i.e., assessing the robustness of the features, was not properly conducted. To achieve optimal feature selection, the slightly better performing feature can be selected from various kinds of similar features via the wavelet transform, which could lead to low reproducibility of wavelet features. Therefore, the stability and reproducibility of selected features must be investigated in further studies.

In conclusion, the present study developed radiomic machine-learning classifiers from multiparametric MR images for the determination of the HPV status in patients with OPSCC. Our results show that logistic regression and the random classifier applied subsequent to feature selection from MR images, including T1WI, T2WI, T1-CEWI, and ADC maps, using LASSO regression exhibit the highest classification accuracy; furthermore, features selected from the ADC map were crucial in classifying the HPV status. This method explores the integration of anatomical and multiparametric MRI radiomics into clinical models, which might have a significant impact in the MR-guided radiotherapy for head and neck cancers.

## Materials and methods

This study was approved by the institutional review board of Asan Medical Center (tertiary referral center). The local ethics committee, institutional review board of Asan Medical Center, waived off the written informed consent due to the retrospective nature of the study. We reported our results according to the standards for reporting of diagnostic accuracy studies (STARD) 2015 guidelines^33^ and strengthening the reporting of observational studies in epidemiology (STROBE)^34^.

### Study population

We enrolled consecutive patients with newly diagnosed histopathologically proved OPSCC, who were examined by head and neck MR imaging between April 2012 and November 2017. The eligibility criteria were as follows: (a) patients diagnosed by histopathology with a pre-treatment OPSCC, (b) patients with known HPV status, (c) patients who were examined by head and neck MR imaging including DWI, and (d) patients that were > 20 years old. Patients who had received chemotherapy, radiation therapy, or excisional biopsy prior to the MR imaging were excluded.

### Analysis of HPV status

All analyses of the HPV status were performed by the pathology division of our institution without prior knowledge of the MR imaging results. P16 immunohistochemistry or HPV DNA detection by polymerase chain reaction (PCR) was used as the reference standard^35,36^. P16 immunohistochemistry was performed using CINtec p16 histology (anti-p16^INK4a^ mouse monoclonal antibody and immunohistochemical detection kit; Roche MTM Laboratories, Heidelberg, Germany) and HPV DNA detection was performed by PCR/DNA chip scanning (high-risk subtypes of 16, 18, 31, 33, 35, 39, 45, 51, 52, 56, 58, 59, 68, 73, 82, and other lower or undetermined risk subtypes)^37^. HPV-positive OPSCC was diagnosed based on the positive results of either p16 or HPV DNA PCR^38^.

### MR acquisition protocol

Head and neck MR imaging was conducted using a 3-T scanner with a 64-channel coil (Skyra, Siemens Healthcare) and the MR imaging protocol as follows: To obtain CE-T1WI, an intravenous dose of 0.1 mmol/kg of contrast agent gadoterate meglumine (Dotarem; Guerbet, Paris, France) was injected into the patient. DWI MR imaging was conducted using multi-shot read-out-segmented echo-planar imaging in the axial plane before the injection. The detailed DWI sequence parameters were as follows: repetition time/echo time, 5450/62 ms; b values of 0 and 1000 s/mm^2^; section thickness of 4 mm; no gap; field of view of 192 $\times$ 192 mm^2^, and acquisition time of approximately 5 min. The ADC maps were obtained automatically within the manufacturer console. Imaging data were de-identified in accordance with the health insurance portability and accountability act privacy rule.

### Image segmentation and pre-processing

Figure 1 depicts the overall workflow. First, 3D ROIs for contrast-enhanced portions were manually segmented by two neuroradiologists (with 6 and 13 years of experience in neuroradiology) on ADC maps for the primary tumour, while also considering T2WI and CE-T1WI MR sequences during the segmentation. One definite pathologically proven malignant lymph node was manually segmented on the T2WI sequence, while also considering CE-T1WI MR sequences. We employed the medical imaging interaction toolkit (MITK) software platform (https://www.mitk.org, German Cancer Research Center, Heidelberg, Germany)^39^. Both the primary tumour and lymph node volumes belonged to the same patient. T1WI, T2WI, CE-T1WI, and ADC maps were co-registered with SPM software (https://www.fil.ion.ucl.ac.uk/spm/), using affine transformation with normalized mutual information as a cost function, with 12 degrees of freedom and tri-linear interpolation^40^. The original ROIs were co-registered on the T1WI, T2WI, and CE-T1WI for the tumour and on the T1WI, CE-T1WI, and ADC maps for the lymph node, then manually adjusted to suit each sequence. All MR images were resampled into isometric voxels of size 1 $\times$ 1 $\times$ 1 mm^3^ as input data. Field inhomogeneity of MR images was corrected using the N4ITK algorithm^41^. To ensure just comparison of the extracted features across all patients, intensity normalization was conducted for T1WI, T2WI, and CE-T1WI sequences.

### Radiomic feature extraction

From the segmented mask, 1618 total radiomic features were extracted using MATLAB R2015a (MathWorks Inc., Natick, MA), using a similar approach to previous study of Yun et al.^42^ at the same institution. The range of mean ± 3 standard deviation of the entire intensity range was quantized into 32 density bin levels for the texture features. The features included seven shape and volume features, 17 first-order features, 162 texture features, and 1432 wavelet features (Supplementary Table 2). First-order features were derived from the intensity histograms using first-order statistics, including the intensity range, energy, entropy, kurtosis, maximum, mean, median, uniformity, and variance. Texture features were obtained from a grey-level co-occurrence matrix (GLCM) and a grey-level run-length matrix (GLRLM) using the segmented mask in 13 directions in 3D space^43^. For the GLCM analyses, texture features were computed for varying distances of 1, 2, and 3 voxels in 13 directions. Then, a single-level directional discrete wavelet transformation was applied with a high-pass and a low-pass filter^44^. In total, eight wavelet-decomposition images were generated from each MR sequence input: LLL, HLL, LHL, HHL, LLH, HLH, LHH, HHH images, where ‘L’ depicts the ‘low-pass filter’ and ‘H’ depicts the ‘high-pass filter’. The first-order and texture features were subsequently applied to the wavelet-transformed images (17 first-order features + 162 texture features) multiplied by eight images, yielding 1432 wavelet features.

### Feature selection and classification

The extracted features may be noisy or highly correlated with each other; therefore, feature selection is required to increase the prediction accuracy and minimise computational cost^45^. To reduce over-fitting or any type of bias in our radiomics model, LASSO-penalized linear regression was applied to the training data. All radiomics features were centred and scaled to a value with a mean of zero and a standard deviation of one (z-score transformation before applying feature selection). With a linear combination of the selected features weighted by their respective coefficients, a model was used to estimate the HPV status. LASSO regression was implemented using Python (Python Software Foundation, version 3.5.2) with the Scikit-learn package (https://github.com/scikit-learn/scikit-learn)^46^. Features with larger contributions to the model were selected.

Three different machine-learning classifiers were applied: logistic regression, random forest^47^ using the Scikit-learn package, and XG boost^48^ using the Xgboost package (https://github.com/dmlc/xgboost). The algorithms were selected based on their high performance and readiness for application. Three different models were computed and compared to determine the best combination for determining the HPV status in the data set. The models were developed separately for each of the T1WI, T2WI, CE-T1WI, and ADC maps, as well as various combinations of these sequences. Classifiers were trained with a stratified threefold cross-validation procedure repeated 20 times, which allows repetition of experiments for each model up to 60 times. All possible combinations of hyperparameters were investigated by the grid search using GridSearchCV library in the Scikit-learn package. (Supplementary Table 3). The feature selector and each classifier were trained with a stratified threefold cross-validation procedure, which was repeated 20 times. This indicates an up to 60-fold repetition of the experiments for each model. The procedures, including z-normalization of extracted features, followed by feature reduction using LASSO regression and machine learning classification were executed separately on the training data during each cross-validation fold.

### Statistical analysis

The Mann–Whitney U test was used to estimate the relationship between selected radiomic signatures and HPV status, and to compare accuracies between various combinations of MR sequences in a pairwise manner^49^. AUCs were used to determine the diagnostic performance, with optimal thresholds of the imaging parameters determined by maximizing the sum of the sensitivity and 1 − specificity, i.e., the Youden index, values.

## Supplementary information

Supplementary Information.

## Data Availability

The datasets generated and analysed during the current study are available from the corresponding author on reasonable request.
