# Supplementary material for: Oropharyngeal squamous cell carcinoma: radiomic machine-learning classifiers from multiparametric MR images for determination of HPV infection status
Source: Sci Rep. 2020 Oct 16;10:17525. doi: 10.1038/s41598-020-74479-x (PMC7568530; doi:10.1038/s41598-020-74479-x)
Supplement: Supplementary file 1 — Supplementary Information. [file 41598_2020_74479_MOESM1_ESM.pdf]

Oropharyngeal squamous cell carcinoma: radiomic machine-learning classifiers from multiparametric MR images for determination of HPV infection status

Chong Hyun Suh, M.D.[1†], Kyung Hwa Lee, M.D. [2,3†], Young Jun Choi, M.D., Ph.D.[1*], Sae Rom Chung, M.D., Ph.D.[1], Jung Hwan Baek, M.D., Ph.D.[1], Jeong Hyun Lee, M.D., Ph.D.[1], Jihye Yun, Ph.D.[1], Sungwon Ham[3], Namkug Kim, Ph.D.[1,4*]

[1]Department of Radiology and Research Institute of Radiology, University of Ulsan College of Medicine, Asan Medical Center, Seoul, Republic of Korea

[2]Department of Medicine, University of Ulsan College of Medicine, Asan Medical Center, Seoul, Republic of Korea

[3]Department of Biomedical Engineering, Asan Medical Institute of Convergence Science and Technology, University of Ulsan College of Medicine, Asan Medical Center, Seoul, Republic of Korea

[4]Department of Convergence Medicine, University of Ulsan College of Medicine, Asan Medical Center, Seoul, Republic of Korea

[†]Both authors contributed equally. (**Co-first authors**)

[*]Both authors contributed equally. (**Co-corresponding authors**)

Supplementary Table 1. Selected features from various combinations of each sequence

Sequence	Selected features			
ADC	ADC	Wavelet_LLL	GLCM_dist_2	Entropy_std
	ADC	Wavelet_HHH	GLCM_dist_3	Correlation
	ADC	Wavelet_HLH	GLCM_dist_3	Correlation
	ADC	Original	GLCM_dist_3	Entropy_std
	ADC	Original	GLCM_dist_1	Cluster Shade std
T1WI	T1	Original	GLCM_dist_1	Autocorrelation_std
	T1	Wavelet_LHL	GLCM_dist_3	Correlation
	T1	Wavelet_LHH	Firstorder	Median
	T1	Wavelet_HLH	GLCM_dist_2	Correlation
	T1	Wavelet_LLH	GLCM_dist_3	Correlation
T2WI	T2	Wavelet_LLH	Firstorder	Median
	T2	Wavelet_LLH	GLCM_dist_1	Cluster Prominence
	T2	Wavelet_HLH	GLRLM	HGRE
	T2	Wavelet_LHH	Firstorder	Skewness
	T2	Wavelet_HLH	GLCM_dist_3	Correlation
CE-T1WI	T1CE	Wavelet_LLH	GLCM_dist_1	Cluster Shade std
	T1CE	Wavelet_LHL	GLCM_dist_1	Cluster Prominence
	T1CE	Wavelet_HLL	GLCM_dist_2	Cluster Prominence std
	T1CE	Wavelet_LLH	Firstorder	Max
	T1CE	Original	Firstorder	Range Cover
ADC+T1WI	ADC	Wavelet_LLL	GLCM_dist_2	Entropy_std
	T1	Original	GLCM_dist_1	Autocorrelation_std
	ADC	Wavelet_HHH	GLCM_dist_3	Correlation
	ADC	Wavelet_HLH	GLCM_dist_2	Correlation_std
	ADC	Wavelet_LLH	GLCM_dist_1	Homogeneity1 std
ADC+T2WI	ADC	Wavelet_LLL	GLCM_dist_2	Entropy_std
	ADC	Wavelet_LLH	GLCM_dist_1	Homogeneity1 std
	ADC	Wavelet_HLH	GLCM_dist_2	Correlation_std
	ADC	Original	GLCM_dist_3	Entropy_std
	T2	Wavelet_HLH	GLRLM	HGRE
ADC+CE-T1WI	ADC	Wavelet_LLL	GLCM_dist_2	Entropy_std
	ADC	Wavelet_HLH	GLCM_dist_2	Correlation_std
	ADC	Wavelet_LLH	GLCM_dist_1	Homogeneity1 std
	ADC	Wavelet_HLH	GLCM_dist_2	Correlation_std
	ADC	Original	GLCM_dist_3	Entropy_std
T1WI+T2WI	T1	Original	GLCM_dist_1	Autocorrelation_std
	T1	Wavelet_LHL	GLCM_dist_3	Correlation
	T2	Wavelet_LLH	GLCM_dist_1	Cluster Prominence std
	T1	Wavelet_LLH	GLCM_dist_3	Correlation
	T1	Original	GLCM_dist_1	Sum average std
T1WI+CE-T1WI	T1	Original	GLCM_dist_1	Autocorrelation_std
	T1	Wavelet_LHL	GLCM_dist_3	Correlation
	T1	Wavelet_LLH	GLCM_dist_3	Correlation
	T1	Original	GLRLM	GLN
	T1	Wavelet_HHL	GLCM_dist_1	Correlation
T2WI+CE-T1WI	T1CE	Wavelet_LLH	GLCM_dist_1	Cluster Shade std
	T2	Wavelet_HLH	GLRLM	HGRE
	T2	Original	Firstorder	Max
	T1CE	Wavelet_LHH	GLCM_dist_1	Cluster Prominence
	T1CE	Wavelet_LHL	GLCM_dist_1	Cluster Prominence
ADC+T1WI+T2WI	ADC	Wavelet_LLL	GLCM_dist_2	Entropy_std
	T1	Original	GLCM_dist_1	Autocorrelation_std
	ADC	Wavelet_HLH	GLCM_dist_2	Correlation_std
	ADC	Wavelet_LLH	GLCM_dist_1	Homogeneity1 std
	ADC	Wavelet_HHH	GLCM_dist_3	Correlation

ADC+T1WI+CE-T1WI	ADC	Wavelet_LLL	GLCM_dist_2	Entropy_std
	T1	Original	GLCM_dist_1	Autocorrelation_std
	ADC	Wavelet_HLH	GLCM_dist_2	Correlation_std
	ADC	Wavelet_LLH	GLCM_dist_1	Homogeneity1 std
	ADC	Wavelet_HHH	GLCM_dist_3	Correlation
ADC+T2WI+CE-T1WI	ADC	Wavelet_LLL	GLCM_dist_2	Entropy_std
	ADC	Wavelet_LLH	GLCM_dist_1	Homogeneity1 std
	ADC	Wavelet_HLH	GLCM_dist_2	Correlation_std
	ADC	Original	GLCM_dist_3	Entropy_std
	ADC	Wavelet_HLH	GLCM_dist_3	Correlation
T1WI+T2WI+ CE-T1WI	T1	Original	GLCM_dist_1	Autocorrelation_std
	T1	Wavelet_LHL	GLCM_dist_3	Correlation
	T1	Wavelet_LLH	GLCM_dist_3	Correlation
	T1	Wavelet_HHL	GLCM_dist_1	Correlation
	T1	Wavelet_LHL	Firstorder	Range Cover

Abbreviations: ADC = apparent diffusion coefficient, T1WI = T1-weighted imaging, T2WI = fat-suppressed T2-weighted imaging, CE-T1WI = fat-suppressed contrast-enhanced T1-weighted imaging, GLCM = gray-level co-occurrence matrix, GLRLM = gray-level run-length matrix.

Supplementary Table 2. Number of extracted features from each sequence

Class	Original	3D Wavelets	Total
First order	17	136	153
GLCM_dist_1	46	368	414
GLCM_dist_2	46	368	414
GLCM_dist_3	46	368	414
GLRLM	24	192	216
Shpae/vol	7	-	7
Total	186	1432	1618

Abbreviations: GLCM = gray-level co-occurrence matrix, GLRLM = gray-level run-length matrix

Supplementary Table 3. Hyperparameters of each classifier

Classifier	Hyperparameters
Logistic regression	C = 0.01<br>penalty = l2<br>class_weight = balanced
Random forest	criterion = gini<br>n_estimators = 300<br>max_features = 1<br>min_sample_split = 2<br>class_weight = balanced
XG boost	objective = binary:logistic<br>n_estimators = 1000<br>max_depth = 1<br>subsample = 0.4<br>colsample_bytree = 0.5

Supplementary Figure 1: Box plots of selected features extracted from four magnetic resonance sequences

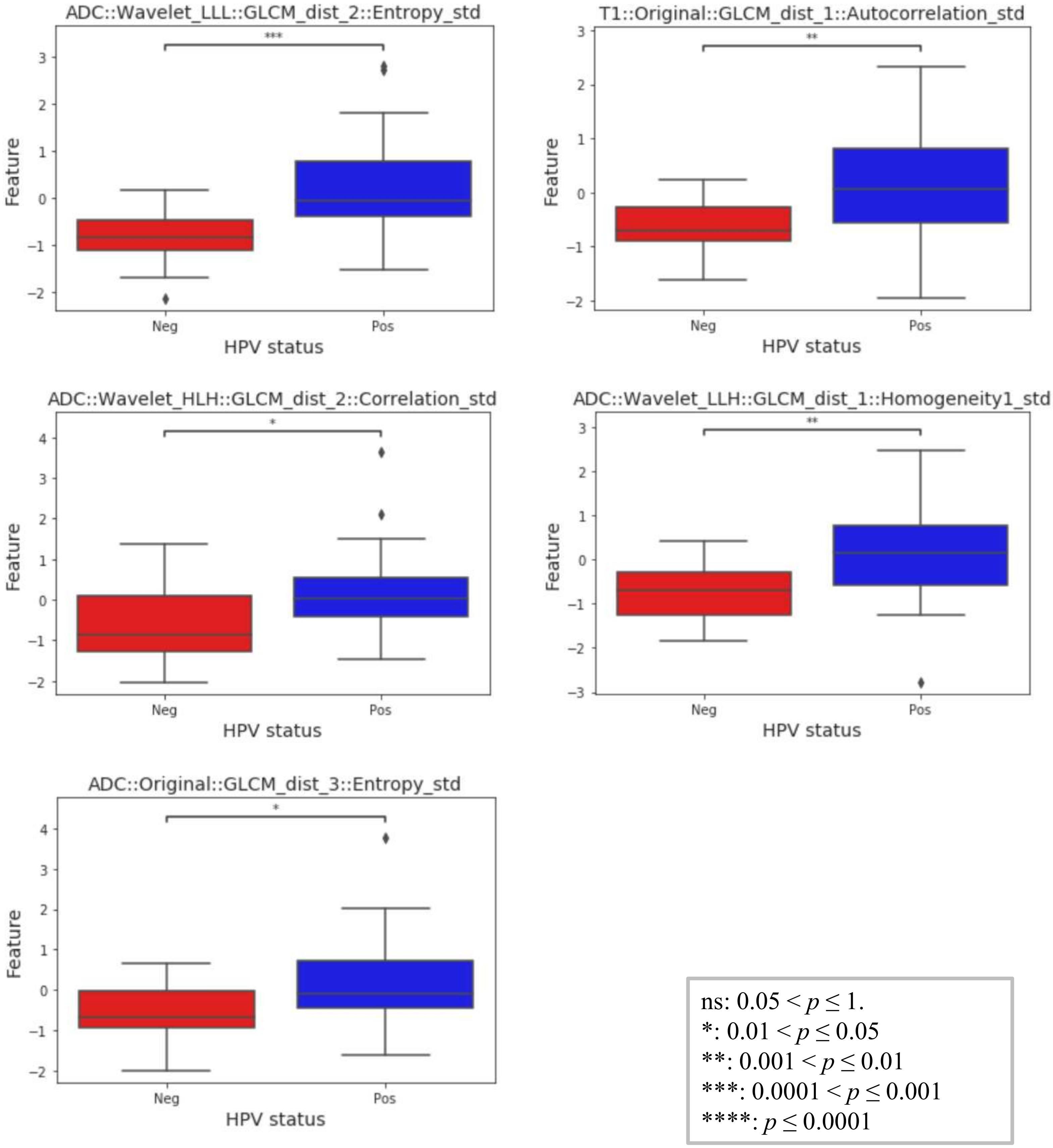

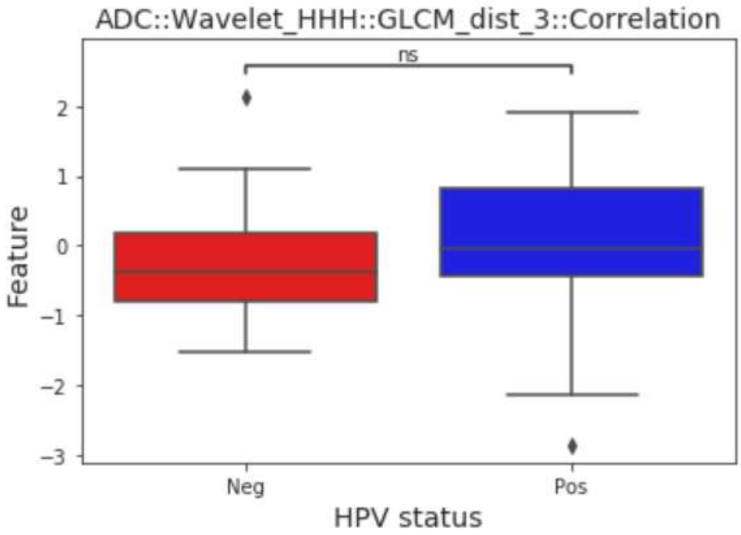

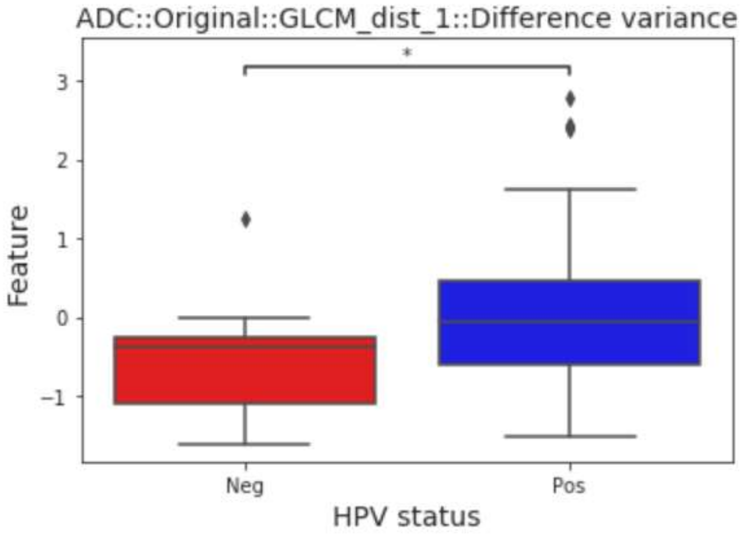

ns: $0.05 < p \leq 1$.
*: $0.01 < p \leq 0.05$
**: $0.001 < p \leq 0.01$
***: $0.0001 < p \leq 0.001$
****: $p \leq 0.0001$

Supplementary Figure 2: Box plots of selected features extracted from ADC map of tumor (T) mask and lymph node (N) mask. (a) Features from T mask. (b) Features from N mask

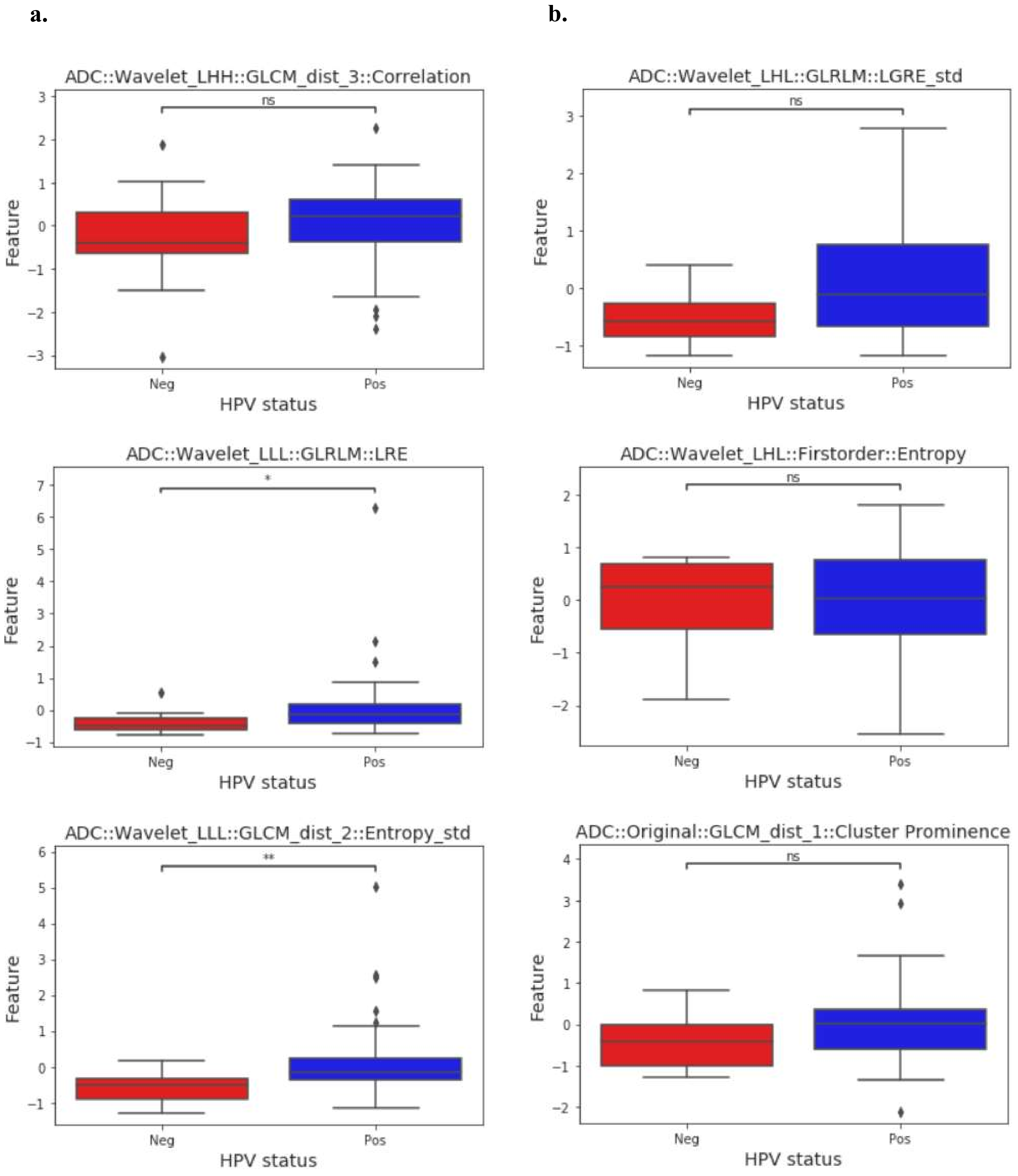

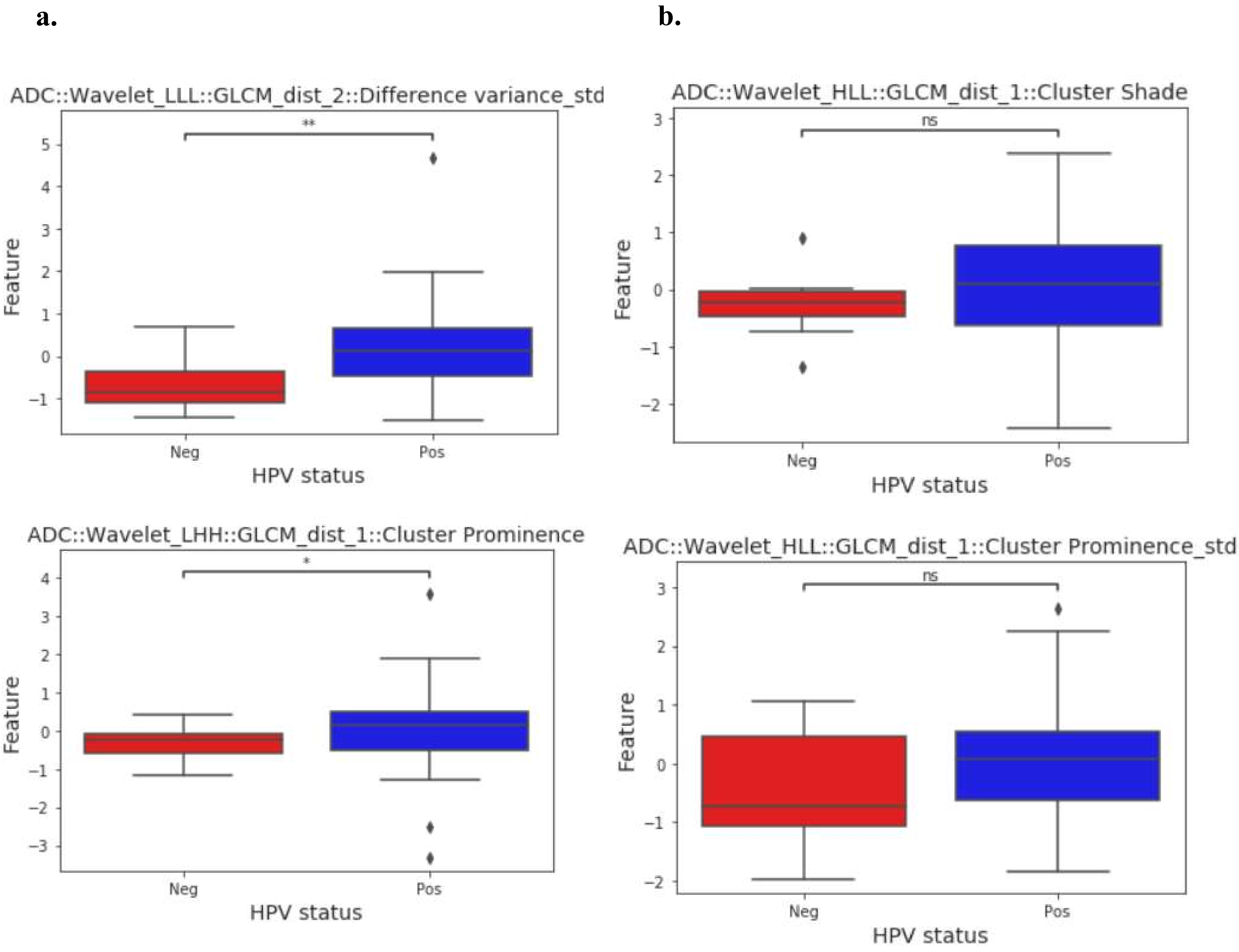

ns: $0.05 < p \leq 1$.
*: $0.01 < p \leq 0.05$
**: $0.001 < p \leq 0.01$
***: $0.0001 < p \leq 0.001$
****: $p \leq 0.0001$
